# Characterization of the complete chloroplast genome of a purple passion flower variety ‘Pingtang No.1’ (*Passiflora edulia* Sims) in China and phylogenetic relationships

**DOI:** 10.1080/23802359.2019.1644235

**Published:** 2019-07-19

**Authors:** Xue-Lian Yang, Xiao-Jing Hu, Xiu-Qin Long

**Affiliations:** a College of Agriculture, Guizhou University, Guiyang, PR China;; b Institute of Guizhou Mountainous Resources, Guiyang, PR China

**Keywords:** *Passiflora edulia* Sims, chloroplast genome, *Passilfora*, phylogenetic relationship analysis

## Abstract

Passion flower (*Passiflora edulia* Sims) is an important fruit that is of great economic importance. In this study, we presented the chloroplast genome of a purple passion flower variety ‘Pingtang No.1’ in China using BGISEQ-500 sequencing. Its chloroplast genome is 152,621 bp in size. It contains a pair of inverted repeat regions of 25,989 bp, each separating a small single copy region of 13,352 bp and a large single copy region of 85,141 bp. Totally, 111 unique genes, including 77 protein coding genes, 30 tRNAs, and 4 rRNAs, were identified and annotated in the chloroplast genome. Phylogenetic maximum likelihood analysis based on chloroplast genomes of 35 plant species, mainly from the genus *Passiflora* indicated that ‘Pingtang No.1’ and yellow passion flower ‘IAPAR-123’ (*Passiflora edulis*) cluster together. The chloroplast genome can be used for a better understanding of the evolutionary relationships of plant species of the family Passifloraceae, especially the genus *Passiflora*.

Passion flower (*Passiflora edulia* Sims), also known as passion fruit, is a fruit with very high nutrient. It is one of the most popular fruits worldwide with great economic importance due to its wide uses in juice production and fresh fruit consumption. Passion flower belongs to the family Passifloraceae. There are more than 700 plant species in the family, most of which belongs to the genus *Passiflora*. Many chloroplast genomes of plant species from *Passiflora* have been reported (Rabah et al. 2019; Shrestha et al. 2019), which is very helpful for the *Pssaiflora* species evolutionary and genetic studies. Passion flower can be divided into yellow passion flower and purple passion flower according to the color of the fruit skin. The chloroplast genome of a yellow passion flower (*Passiflora edulia*) has also been reported in 2017 (Cauz-Santos et al. 2017). Up to now, however, the genome chloroplast genome sequence of purple passion flower is still not very clear. In this study, the chloroplast genome of a purple passion flower variety ‘Pingtang No.1’ was assembled and characterized using BGISEQ-500 sequencing data and its relationship within the genus *Passiflora* was also explored.

The specimen sample of ‘Pingtang No.1’ was isolated from Liujiawan village, Kedu Town, Pingtang County, Guizhou Province, China (25°38′27″N; 106°52′38″E) and the sample was deposited at Guizhou University. The total genomic was extracted from fresh leaves according to Chen et al. (2019) and stored at the Guizhou University (No. GZUPT01). The total genomic DNA was used for the shotgun library construction and sequenced using BGISEQ-500 sequencing platform to generate about 1 Gbp data. The obtained high quality reads were first aligned to the chloroplast genomes of plant species from the genus *Passiflora*, including *Passiflora edulis* (KX290855.1), *Passiflora cincinnata* (KY820583.1), *Passiflora actinia* (MF807934.1), *Passiflora auriculata* (MF807936.1), *Passiflora biflora* (MF807937.1), *Passiflora laurifolia* (MF807939.1), *Passiflora ligularis* (MF807940.1), *Passiflora nitida* (MF807941.1), *Passiflora oerstedii* (MF807942.1), *Passiflora pittieri* (MF807943.1), *Passiflora quadrangularis* (MF807944.1), *Passiflora retipetala* (MF807945.1), *Passiflora serratodigitata* (MF807946.1), *Passiflora vitifolia* (MF807947.1), *Passiflora serratifolia* (MF807948.1), *Passiflora jatunsachensis* (MK694920.1), *Passiflora suberosa* (MK694921.1), *Passiflora lutea* (MK694922.1), *Passiflora tenuiloba* (MK694923.1), *Passiflora rufa* (MK694924.1), *Passiflora contracta* (MK694925.1), *Passiflora arbelaezii* (MK694926.1), *Passiflora tetrandra* (MK694927.1), *Passiflora misera* (MK694928.1), *Passiflora filipes* (MK694929.1), *Passiflora affinis* (K694930.1), *Passiflora obovate* (MK694931.1), *Passiflora foetida* (MK694932.1), *Passiflora menispermifolia* (MK694933.1), and *Passiflora microstipula* (MK694934.1). The chloroplast genome was then assembled using CLC Genomics Workbench v8.0 (CLC Bio, Aarhus, Denmark), annotated using DOGMA (Wyman et al. 2004) and Geneious (Kearse et al. 2012). The annotated chloroplast genome has been deposited in Genbank with the accession number MN099051.

The complete chloroplast genome of ‘Pingtang No.1’ is 152,621 bp in size, containing a large single copy region of 85,141 bp and a small single copy region of 13,352 bp that is separated by a pair of inverted repeat regions of 25,989 bp. The chloroplast genome contains 111 unique genes, including 77 protein-coding genes, 30 tRNAs, and 4 rRNAs. A total of 19 genes, including eight protein coding genes (i.e. *ycf2*, *ycf1*, *rps12*, *rps7*, *rps19*, *rpl23*, *rpl2,* and *ndhB*), 7 tRNAs (i.e. *trnV-GAC*, *trnR-ACG*, *trnN-GUU*, *trnL-CAA*, *trnI-GAU*, *trnI-CAU*, and *trnA-UGC*) and 4 rRNAs (*4.5S*, *5S*, *16S,* and *23S* rRNAs) were found duplicated in the inverted repeat regions. The overall nucleotide composition of the chloroplast genome is: 30.6% A, 31.5% T, 18.6% C, and 17.8% G, with the total GC content of 36.5%.

For phylogenetic maximum likelihood analysis, we downloaded the chloroplast genomes of 30 plant species belonging to Passifloraceae and 3 plant species from Actinidiaceae (as outgroup) from GenBank to access the relationship of ‘Pingtang No. 1’ with them. Chloroplast genome sequences were aligned using HomBlocks pipeline (Bi et al. 2018). RAxML-HPC2 on XSEDE version 8.2.10 (Stamatakis 2014) was used to construct a maximum likelihood tree, for which 1000 bootstrap replicates were used to calculate the bootstrap values. Result showed that ‘Pingtang No.1’ and yellow passionflower ‘IAPAR-123’ (*Passiflora edulis*) cluster together, indicating that their relationship was the closest (Figure 1). By comparing their chloroplast genome sequences, we found that, compared with ‘IAPAR-123’, the chloroplast genome of ‘Pingtang No.1’ was larger, with more unique protein-coding genes, and more notable is its longer inverted repeat region. The chloroplast genome can be subsequently used for a better understanding of the plant species evolutionary relationships within the family Passifloraceae, especially within the genus *Passiflora*.

**Figure 1.**
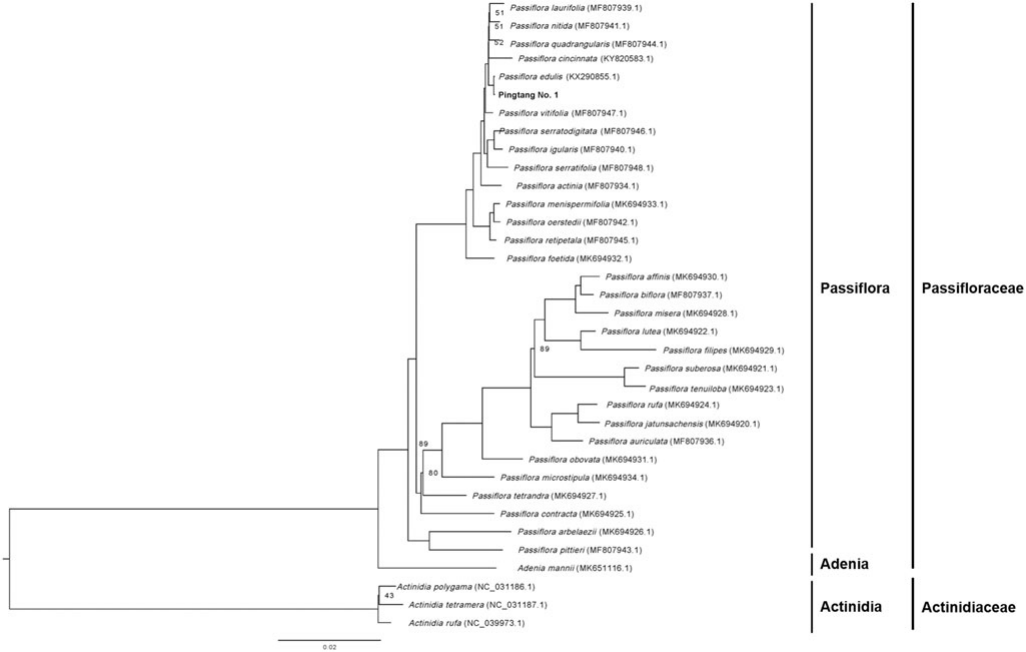
Maximum likelihood phylogenetic tree based on the complete chloroplast genome sequences of 34 plant species with *Actinidia polygama*, *Actinidia tetramera,* and *Actinidia rufa* as outgroup. Numbers on the nodes are bootstrap values with 1000 replicates and bootstrap values of 100 were omitted.
